# Z-Type Heterojunction MnO_2_@g-C_3_N_4_ Photocatalyst-Activated Peroxymonosulfate for the Removal of Tetracycline Hydrochloride in Water

**DOI:** 10.3390/toxics12010070

**Published:** 2024-01-14

**Authors:** Guanglu Lu, Xinjuan Li, Peng Lu, He Guo, Zimo Wang, Qian Zhang, Yuchao Li, Wenbo Sun, Jiutao An, Zijian Zhang

**Affiliations:** 1 College of Resources and Environment Engineering, Shandong University of Technology, Zibo 255000, China; lgl8899168@163.com (G.L.); 15908037096@163.com (X.L.); 15834482173@163.com (P.L.); zq@sdut.edu.cn (Q.Z.); wenbosun@sdut.edu.cn (W.S.); 2 Department of Environmental Engineering, College of Biology and the Environment, Nanjing Forestry University, Nanjing 210037, China; heguo@njfu.edu.cn; 3 Department of Marine Engineering, Jimei University, Xiamen 361021, China; wangzimo@163.com; 4 Research Institute of Clean Chemical Technology, School of Chemistry and Chemical Engineering, Shandong University of Technology, Zibo 255049, China; cyulee@126.com

**Keywords:** MnO_2_@g-C_3_N_4_, peroxymonosulfate, photocatalytic, degradation mechanism, TC

## Abstract

A Z-type heterojunction MnO_2_@g-C_3_N_4_ photocatalyst with excellent performance was synthesized by an easy high-temperature thermal polymerization approach and combined with peroxymonosulfate (PMS) oxidation technology for highly efficient degrading of tetracycline hydrochloride (TC). Analysis of the morphological structural and photoelectric properties of the catalysts was achieved through different characterization approaches, showing that the addition of MnO_2_ heightened visible light absorption by g-C_3_N_4_. The Mn_1_-CN_1_/PMS system showed the best degradation of TC wastewater, with a TC degradation efficiency of 96.97% following 180 min of treatment. This was an approximate 38.65% increase over the g-C_3_N_4_/PMS system. Additionally, the Mn_1_-CN_1_ catalyst exhibited excellent stability and reusability. The active species trapping experiment indicated •OH and SO_4_^•−^ remained the primary active species to degrade TC in the combined system. TC degradation pathways and intermediate products were determined. The Three-Dimensional Excitation-Emission Matrix (3DEEM) was employed for analyzing changes in the molecular structure in TC photocatalytic degradation. The biological toxicity of TC and its degradation intermediates were investigated via the Toxicity Estimation Software Test (T.E.S.T.). The research offers fresh thinking for water environment pollution treatment.

## 1. Introduction

Tetracycline antibiotics are among the most frequently adopted antibiotics in livestock, aquaculture, and healthcare [1]. Tetracycline hydrochloride (TC), a tetracycline antibiotic, cannot be completely broken down in the environment because of its stable chemical properties [2]. The misuse and release of antibiotics in the ecosystem can promote the development of various drug-resistant bacteria and the enhancement of resistance genes [3], causing severe risk to global health and the ecosystem [4,5]. As a result, it is important to find a green, effective, and economical way to degrade TC effluent.

Currently, the commonly used remediation methods for environmental pollutants include adsorption [6], flocculation precipitation [7], ion exchange [8], and biochemical methods [9,10]. However, the above methods have the disadvantages of complicated procedures, high costs, and low removal efficiency. Advanced oxidation processes (AOPs) are considered a hopeful degradation technology because of their ability to generate highly reactive radicals, including hydroxyl radicals (•OH) and sulfate radicals (SO_4_^•−^) [11,12]. Interestingly, peroxymonosulfate (PMS)-based AOPs (PMS-AOPs) can produce SO_4_^•−^ with higher redox potential, better selectivity, and a more comprehensive pH selection range [13]. The PMS could be excited by photocatalysis, carbon materials, and transition metal oxides to generate SO_4_^•−^ [13,14,15,16,17]. Photocatalysis activation of PMS is one of the most widely used techniques because photocatalytic methods are inexpensive, simple, and efficient [18]. Many scholars have reported the effective degradation of pollutants by combining photocatalysts with PMS activation [15]. Liu et al. [19] found that the combination of graphitic carbon nitride (g-C_3_N_4_) nanosheets and PMS enhanced the removal of Bisphenol A under visible light, with complete removal in 90 min. Guan et al. [20] investigated whether the introduction of persulfate in the Co_3_O_4_/CeO_2_ system improved the removal efficacy of TC. Therefore, the combination of PMS and photocatalytic systems can effectively degrade antibiotic wastewater.

Recently, with good stability, reaction to light in the visible range, and unique properties of affordability, g-C_3_N_4_ has gained much attention as a non-metallic semiconductor material [21]. Meanwhile, research has now demonstrated that g-C_3_N_4_ can activate PMS to degrade organic pollutants [19]. Unfortunately, g-C_3_N_4_ still has significant limitations, including weak conductivity and quick recombination of photogenerated electron-holes (e^−^-h^+^), which restricts its practical applicability in environmental purification [22]. Hence, numerous techniques have been developed for improving the photocatalytic activity of g-C_3_N_4_, involving metal or non-metal doping [23,24], construction nanostructures [25], and combinations of different semiconductors [26]. Among all modification methods, the combination of other semiconductors and g-C_3_N_4_ remains an excellent method for constructing Z-type heterojunction materials [27,28].

Transition metal oxide semiconductors can serve as catalysts and PMS activators to produce strongly oxidizing SO_4_^•−^. As a typical transition metal oxide, manganese dioxide (MnO_2_) has the benefits of low cost, environmental friendliness, and good conductivity [29]. MnO_2_ is an effective PMS activator due to the abundant oxygen mobility in the MnO_2_ lattice and the electron transfer between Mn species [30,31]. Therefore, the addition of MnO_2_ to g-C_3_N_4_ for forming a Z-type heterojunction is a viable approach for improving photocatalytic performance of the catalyst [32,33].

In our research, a MnO_2_@g-C_3_N_4_ heterojunction with a Z-type structure was prepared for efficient TC photocatalytic degradation with PMS and visible light. Various characterization approaches were employed for analyzing the morphology, chemical compositions, and optoelectronic properties of samples. The photocatalytic degradation performance of TC was studied under diverse reaction environment parameters. Meanwhile, the intermediates of TC and possible reaction pathways were determined. The Toxicity Estimation Software Test (T.E.S.T.) was employed for assessing TC toxicity and its intermediates. The average TC removal rate observed under the current procedures at the wastewater treatment plants was 96.97%. This research presents a safe, green, and environmentally friendly treatment method for purifying and treating antibiotic wastewater.

## 2. Experimental Section

### 2.1. Materials

The chemical reagents used in our research are all listed in Supplementary file Text 1.

### 2.2. Photocatalyst Sample Preparation

#### 2.2.1. Preparation of g-C_3_N_4_

The g-C_3_N_4_ preparation had two steps. In the first step, bulk g-C_3_N_4_ was produced through thermal polycondensation [34]. A total of 5 g of melamine was laid in a muffle furnace, with the temperature heated to 550 °C at 5 °C min^−1^ and held for 4 h. After cessation of heating, the yellowish solid was ground for 30 min to form a fine powder. To pyrolytically peel the prepared bulk g-C_3_N_4_ into thinner g-C_3_N_4_, in the second step, the yellowish g-C_3_N_4_ powder was divided into several portions, and the g-C_3_N_4_ was laid in crucible and placed in a muffle furnace, with the temperature heated to 500 °C at 10 °C min^−1^ within 2 h to obtain g-C_3_N_4_.

#### 2.2.2. Preparation of MnO_2_

MnO_2_ was synthesized by redox coprecipitation using MnCl_2_-4H_2_O and KMnO_4_ (molar ratio 3:2). The brown precipitate was separated by adding KMnO_4_ solution (2 mol L^−1^ 100 mL) to MnCl_2_ solution (3 mol L^−1^ 100 mL) dropwise at 5 mL min^−1^ during magnetic stirring and drying for 4 h at 90 °C. The solution was stirred again to redissolve the dried powder in water. The precipitate was washed properly with alcohol by filtration and dried for 12 h at 90 °C to obtain MnO_2_.

#### 2.2.3. Preparation of MnO_2_@g-C_3_N_4_

Three mass ratios of MnO_2_ and g-C_3_N_4_ (MnO_2_:g-C_3_N_4_ = 1:2, 1:1, and 2:1) were weighed and were recorded as Mn_1_-CN_2_, Mn_1_-CN_1_, and Mn_2_-CN_1_, respectively. The mixture was ground for 30 min to form a fine powder and then laid in crucible in a muffle furnace, with the temperature heated to 400 °C at 5 °C min^−1^ within 4 h and chilled to normal temperature to obtain Mn-CN photocatalytic composite materials with different ratios.

### 2.3. Photocatalyst Characterization

The morphology and dimensions of the photocatalysts were identified using scanning electron microscopy (SEM) techniques. X-ray diffraction (XRD) patterns were obtained using a Brucker D8 Advance diffraction apparatus. The surface chemistry of the samples was characterized by X-ray photoelectron spectroscopy (XPS). Solid-state UV-Vis diffuse reflectance spectroscopy (UV-Vis-DRS) was used to study the optical properties and separation of photogenerated electrons and holes. Electrochemical impedance spectroscopy (EIS) was measured using a CHI 660B electrochemical system. The degradation intermediates of TC were analyzed by HPLC-MS. The molecular structural changes during TC degradation were analyzed using Three-Dimensional Excitation-Emission Matrix (3DEEM). The parameters used for all characterizations are given in Supplementary file Text 2.

### 2.4. Photocatalytic Degradation of TC by Mn-CN

In the experiments, the photocatalytic degradation performance of the catalysts was tested via a dominant wavelength of 400 nm and a 300 W solar simulator xenon lamp (xenon lamp model and manufacturer’s declared spectrum as well as photocatalytic device diagram are shown in Supplementary Figure S1). The photocatalyst (0.03 g) was dispersed into 50 mL of 20 mg L^−1^ TC solution for each experiment. The solution was stirred for 30 min without light until adsorption equilibrium was reached. Then, 0.6 mM PMS was added, and the lamp was switched on. Samples were taken every 30 min during the photocatalytic process. Supernatant measurement was performed with UV-Vis spectrophotometer (UV-5100B) at 357 nm after filtration through a 0.22 μm microfilter membrane.

## 3. Results and Discussion

### 3.1. Properties of the Material

**Figure 1:**
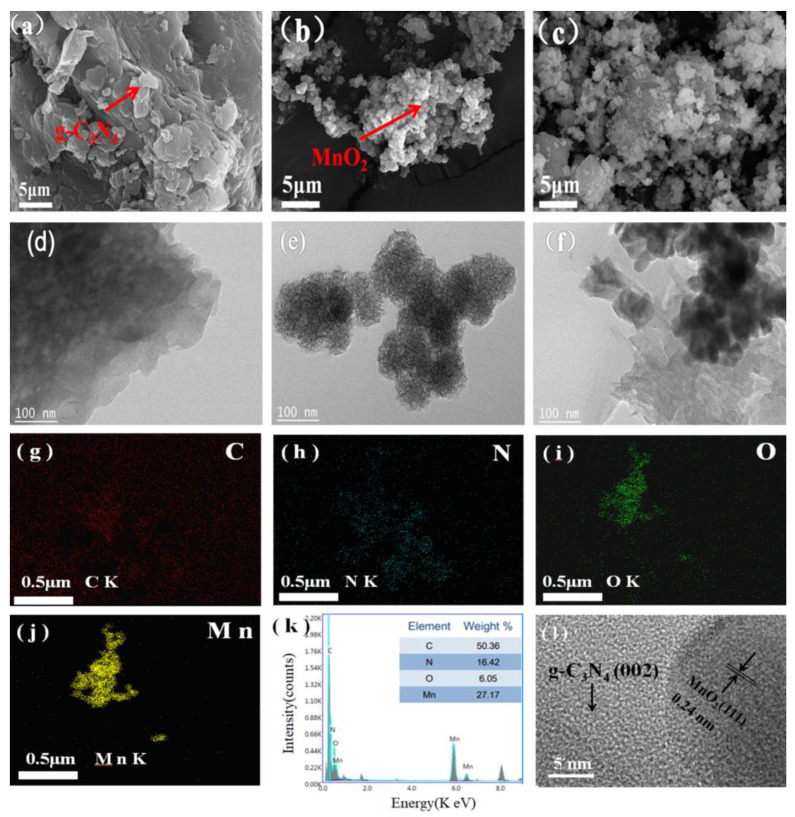
SEM and TEM pictures of (**a**,**d**) g-C_3_N_4_, (**b**,**e**) MnO_2_, (**c**,**f**) Mn_1_-CN_1_; (**g**–**j**) elemental mapping images of C, N, O, and Mn in Mn_1_-CN_1_; (**k**) EDS spectrum of Mn_1_-CN_1_; (**l**) HRTEM image of Mn_1_-CN_1_.

Figure 1 displays the SEM and TEM pictures of the samples. The g-C_3_N_4_ was composed of many irregular lamellar and layered structures [35], with a large specific surface area, as shown in Figure 1a [36]. MnO_2_ exhibited regular small particle-size nanospheres with a rough surface but a relatively uniform particle size distribution (Figure 1b). Moreover, MnO_2_ was dispersed on the g-C_3_N_4_ layered structure in Mn_1_-CN_1_ (Figure 1c). Figure 1d–f shows the TEM images of the sample. Many MnO_2_ particles were found on the g-C_3_N_4_ surface, indicating that the composites of MnO_2_ and g-C_3_N_4_ were well prepared [37]. As depicted in Figure 1g–j, C, N, O, and Mn were evenly scattered on the Mn_1_-CN_1_ surface. The percentages of C, N, O and Mn in Mn_1_-CN_1_ were 50.36%, 16.42%, 6.05%, and 27.17%, respectively (Figure 1k). Figure 1l shows the HRTEM image of Mn_1_-CN_1_; two different types of lattice stripes can be found from the (002) and (111) facets of the crystal structure at crystallographic plane distances of approximately 0.34 and 0.24 nm, respectively.

**Figure 2:**
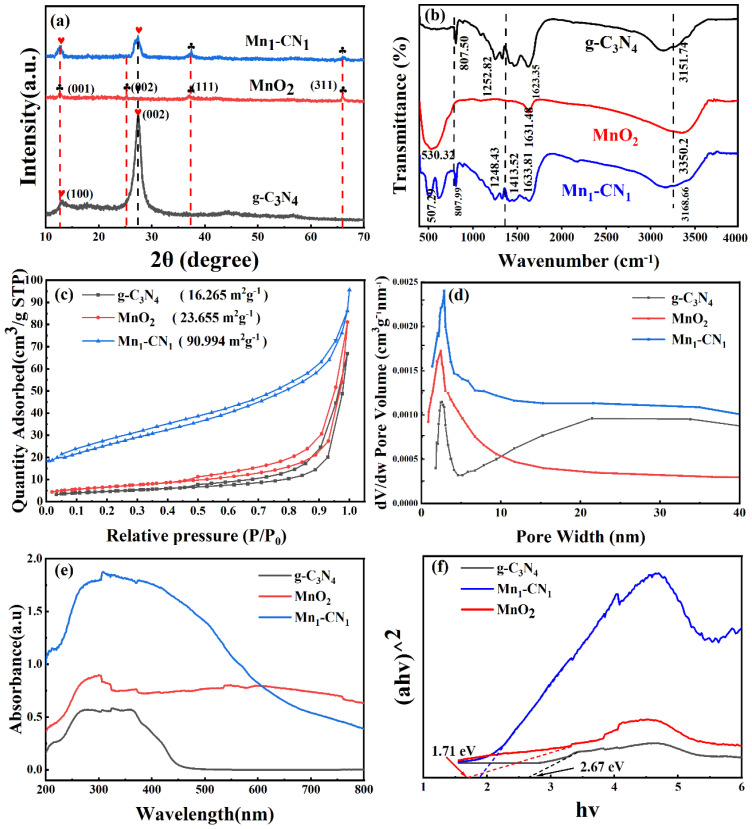
(**a**) XRD patterns (The hearts and clubs in 2a are the locations of the g−C_3_N_4_ and MnO_2_ characteristic peaks, respectively), (**b**) FT-IR spectra, (**c**) N_2_ adsorption-desorption isotherm curve, (**d**) pore size distribution of g-C_3_N_4_, MnO_2_, and Mn_1_-CN_1_, (**e**) UV-vis DRS spectra of the samples, (**f**) diffuse reflectance spectra after Tauc fitting for the different photocatalysts.

Figure 2a displays the XRD patterns of g-C_3_N_4_, MnO_2_, and Mn_1_-CN_1_. The g-C_3_N_4_ and Mn_1_-CN_1_ have the same peaks located at 13.1° and 27.4° [38]. The diffraction peak at 13.1° was weak and belonged to the (100) crystal plane of g-C_3_N_4_, which was made up of repeating structural units of the heptazine ring. The diffraction peak at 27.4° was strong and belonged to the g-C_3_N_4_ (002) crystal plane. This indicates that the sample was a lamellar stacking of graphite-like material [39]. The location and intensity of MnO_2_ diffraction peaks were relatively uniform and smooth, with the diffraction peaks at 2θ = 12.7°, 25.2°, 37.1°, and 66.0° for lattice planes (001), (002), (111), and (311), respectively [40]. The relatively weak intensity and broad peak pattern of these diffraction peaks indicated that the product was a compact growth of amorphous and weakly crystalline MnO_2_. Furthermore, all prominent peaks of g-C_3_N_4_ and MnO_2_ could be seen in the Mn_1_-CN_1_ spectra, which indicates that MnO_2_ was compounded into g-C_3_N_4_ to form Mn_1_-CN_1_.

Figure 2b displays the FT-IR of the sample. The peak of g-C_3_N_4_ at around 807.50 cm^−1^ can be related to the triazinic moiety [41]. Vibrational absorption peaks at 1252.82 and 1623.35 cm^−1^ correspond to the vibrational stretch of the carbon and nitrogen bonds (C-N and C=N). The stretching vibrations of -NH_2_ and -NH groups and of the -OH bond correspond to the peak at 3151.74 cm^−1^ [42]. The characteristic peak of MnO_2_ was mainly at 530.32 cm^−1^. The positions of the Mn_1_-CN_1_ absorption peaks were essentially the same as those of MnO_2_ and g-C_3_N_4_ absorption peaks, illustrating that the MnO_2_ had been loaded onto g-C_3_N_4_.

Samples’ N_2_ adsorption-desorption isotherms and pore-size profiles were measured, with the results shown in Figure 2c,d. The specific surface area of Mn_1_-CN_1_ was 90.99 m^2^g^−1^, which was 5.6 and 3.8 times higher relative to g-C_3_N_4_ and MnO_2_, respectively. Due to the large number of MnO_2_ particles tightly dispersed on the surface of g-C_3_N_4_, the specific surface area of the composites was enlarged relative to single g-C_3_N_4_ and MnO_2_, which contributed to TC degradation. Under the International Union of Pure and Applied Chemistry (IUPAC) classification, all three samples displayed the Type IV isotherm of the H_3_ type, indicating that narrow pores existed, unique to mesoporous materials, in the prepared samples [43]. From Figure 2d, the pore size distribution curves of all photocatalysts, g-C_3_N_4_, MnO_2_, and Mn_1_-CN_1_, indicate a mesoporous structure. Moreover, the pore volume of Mn_1_-CN_1_ was also greater relative to g-C_3_N_4_ and MnO_2_. The larger specific surface area and higher porosity of the catalyst could increase the contact area of active molecules and pollutants, thus enhancing photocatalytic performance [44].

The UV-Vis DRS spectrum shows the optical properties of samples (Figure 2e). The G-C_3_N_4_ sample absorbed light within the range of 450 nm to UV. Compared with g-C_3_N_4_, MnO_2_ exhibited strong absorption in the wavelength range of 300–800 nm. Notably, the introduction of MnO_2_ resulted in a significant red-shift of Mn_1_-CN_1_, indicating that the MnO_2_ was able to extend the absorption range of Mn_1_-CN_1_ for visible light [45]. The bandgap energy of the samples was calculated via the Kubelka–Munk function [46], as shown below:

(1) $${\left(\alpha hv\right)}^{1/2}=K\left(hv-E_{g}\right)$$

where *α*, *v*, *h*, *K*, and *E_g_* are absorbance, photon frequency, Planck’s constant, proportional constant, and bandgap energy, respectively. This can be derived from the diffuse reflectance spectra after Tauc fitting with *E_g_* values of 2.67 eV and 1.71 eV for g-C_3_N_4_ and MnO_2_ samples, respectively (Figure 2f). Valence bands (*VB*) of g-C_3_N_4_ and MnO_2_ can be estimated from the XPS valence band spectra (Supplementary Figure S2), with positions of 1.54 and 2.34 eV, respectively. The conduction band (*CB*) position was calculated via Equation (2), and the *CB* potentials of g-C_3_N_4_ and MnO_2_ were −1.13 and 0.63 eV, respectively.

(2) $$E_{CB}=E_{VB}-E_{g}$$

**Figure 3:**
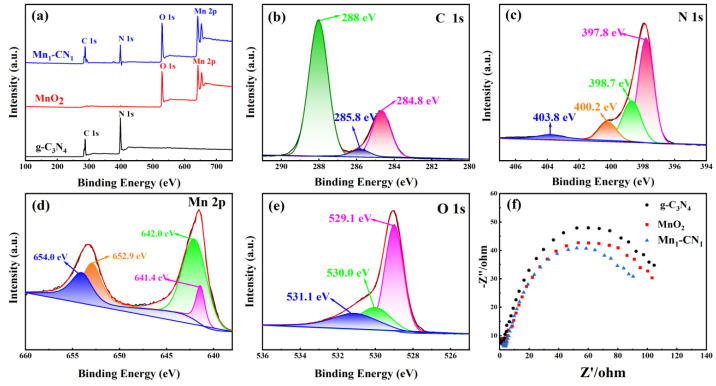
Extended range XPS spectra of g-C_3_N_4_, MnO_2_, and Mn_1_-CN_1_ (**a**); high-resolution XPS patterns of C 1s (**b**), N 1s (**c**), Mn 2p (**d**), and O 1s (**e**); electrochemical impedance spectra (**f**).

Figure 3a displays the total XPS spectra of the photocatalysts. The XPS spectrum of Mn_1_-CN_1_ contained peaks of C 1s and N 1s elements in g-C_3_N_4_ and peaks of Mn 2p and O 1s elements in MnO_2_. In addition, no peaks of other elements were found in the Mn_1_-CN_1_ catalyst, indicating that the composite was not doped with impurities of other elements. Figure 3b shows the three peaks obtained by fitting the C 1s high resolution spectrum with the XPSPEAK software. The three peaks at 284.2, 285.2, and 287.4 eV resulted from the C-C bond, C=N bond, and N=C-N_2_ group, respectively [47]. In Figure 3c, the four characteristic peaks of N 1s spectra at 397.8, 398.7, 400.2, and 403.8 eV originated from the C=N-C bond of sp^2^-hybridized aromatic nitrogen, (C)_3_-N_2_ bond of the sp^2^-hybridized bridged nitrogen atom, C-N-H of the sp^3^-hybridized terminal functional group [48,49], and the charge effect of the π-excited states [50], respectively. These results prove the existence of the graphite phase g-C_3_N_4_. The two characteristic peaks in Mn 2p spectra (Figure 3d) at 642.0 and 654.0 eV corresponded to the binding energy spin-orbit double peaks of Mn 2p^3/2^ and Mn 2p^1/2^, respectively, which is consistent with the nature of MnO_2_. In addition, the change in the valence of Mn was crucial for the photocatalytic reaction. The presence of characteristic peaks at 652.9 and 641.4 eV indicates that both Mn^3+^ and Mn^4+^ were present in MnO_2_. As shown in the O 1s spectra (Figure 3e), the three peaks at 529.1, 530, and 531.1 eV were due to the atomic lattice oxygen of MnO_2_, hydroxyl groups attached to the Mn surface, and adsorbed oxygen [51].

In addition, the interfacial charge transfer of the samples was studied using the EIS technique. Photoelectric performance tests were performed on a CHI 660B electrochemical system. The system used a standard three-electrode system with a photocatalyst as the working electrode, a Pt plate counter electrode and an Ag/AgCl reference electrode, and Na_2_SO_4_ as the electrolyte solution. The smaller arc radius of the EIS curve indicates lower resistance of the sample and a better electron-hole pair separation [52]. From Figure 3f, it can be seen that the electrochemical impedance arc radius of Mn_1_-CN_1_ was significantly smaller relative to g-C_3_N_4_ and MnO_2_, indicating that Mn_1_-CN_1_ possessed the smallest charge transfer resistance and quicker interfacial charge transfer efficiency. The above results show that the formation of heterojunctions could speed up the transfer of photo-excited electron-hole pairs, which improved photocatalytic degradation [28].

### 3.2. Photocatalytic Efficiency of the Samples toward TC Degradation

**Figure 4:**
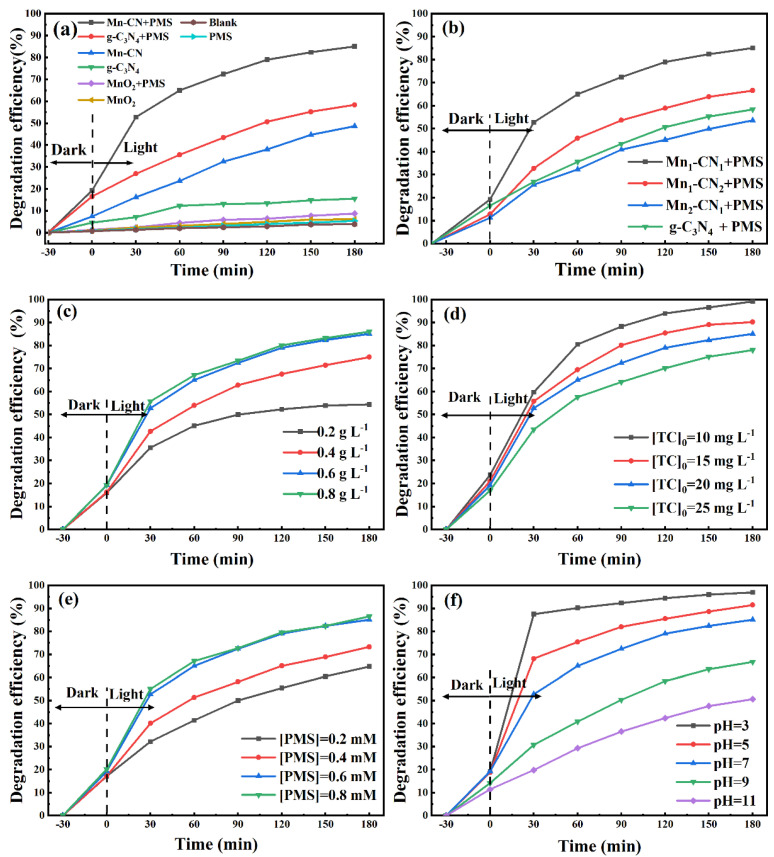
Photocatalytic degradation efficiency of (**a**) the different photocatalysts, (**b**) Mn-CN in different proportions, (**c**) amounts of the photocatalyst, (**d**) initial TC concentration, (**e**) PMS dosage, and (**f**) initial pH. The reaction conditions: [TC] = 20 mg L^−1^, [photocatalyst] = 0.6 g L^−1^, [PMS] = 0.6 mM, T = 25 °C, and pH = 7.

From Figure 4a, it can be seen that the Mn-CN/PMS system exhibited excellent degradation performance, with a degradation efficiency of 85.06%. When MnO_2_ was introduced, g-C_3_N_4_/MnO_2_ samples displayed excellent photocatalytic degradation efficiency for TC, which resulted from the formation of the g-C_3_N_4_/MnO_2_ heterojunction. However, degradation efficiency of TC for the sole MnO_2_ system was less than 7.0%. Moreover, the TC degradation efficiency for the sole g-C_3_N_4_ or PMS system was also relatively low, with only 15.5% and 5.4% removal, respectively. When both Mn-CN and PMS were present, the TC degradation efficiency reached 85.06%, suggesting that PMS was activated by the Mn-CN activator.

The photocatalytic degradation performance of different Mn-CN catalysts is shown in Figure 4b. Compared with other samples, the Mn_1_-CN_1_ catalysts exhibited better degradation performance. TC degradation efficiency achieved 85.06% when PMS existed after 180 min. However, when the MnO_2_ ratio increased, the TC degradation efficiency did not significantly change because the excessive MnO_2_ increased the absorption and scattering of photons, which affected light utilization by g-C_3_N_4_ and reduced photocatalytic efficiency. This is consistent with previous reports [23].

### 3.3. The Effect of Catalyst Dosage

TC degradation efficiency was enhanced when the amount of the Mn_1_-CN_1_ catalyst increased (Figure 4c). When the Mn_1_-CN_1_ catalyst was dosed from 0.2 to 0.6 g L^−1^, TC degradation efficiency improved from 54.3% to 85.06%. With the increase in the amount of the Mn_1_-CN_1_ catalyst, the number of active sites increased, which was helpful for improving TC photocatalytic degradation efficiency [26]. However, TC degradation efficiency was not significantly improved by more than 1% when the Mn_1_-CN_1_ dose reached 0.8 g L^−1^. When the amount of the Mn_1_-CN_1_ catalyst was too high, the excessive Mn_1_-CN_1_ impeded the passage of light, further affecting the number of photogenerated electron-hole pairs generated by the photocatalyst and causing the degradation efficiency of TC solution to decrease.

### 3.4. The Effect of initial TC Concentration

As Figure 4d displays, as the TC concentration was augmented from 10 to 25 mg L^−1^, the TC degradative efficiency diminished from 99.1% to 77.9% in 180 min photoreaction. When the catalyst and the PMS dosage were constant, the electron-hole pairs produced were almost constant, and the number of active molecules produced by the photocatalytic system tended to be constant. The photocatalytic system was able to provide sufficient active substances while the initial TC concentration was 10 mg L^−1^. Therefore, each TC molecule was more easily oxidized, improving the utilization rate of the active species. The photocatalytic system provided insufficient active species, and TC degradation efficiency diminished, with gradual augmentation of the initial TC concentration. In addition, the amount of intermediate products produced in TC degradation also increased with the initial TC concentration. This could result in occupation of other active sites [28].

### 3.5. The Effect of PMS Dosage on TC Degradation

The influence of different PMS concentrations on TC degradation is detailed in Figure 4e. As the PMS dosage was augmented, the TC degradation efficiency was gradually enhanced. The photocatalytic degradation efficiency of TC increased from 64.7% to 85.06% when the concentration of PMS was increased from 0.2 to 0.6 mM, which was attributed to the production of more reactive oxygen species by Mn_1_-CN_1_ [53]. However, the TC degradation capacity was not significantly improved when PMS was dosed to 0.8 mM, with no great improvement in TC degradation efficiency. Excess PMS could react with SO_4_^•−^, forming persulfate radicals (S_2_O_8_^2−^), which have a much lower oxidation capacity than SO_4_^•−^ (see Equations (3) and (4)) [54].

(3) $${\mathrm{SO}}_{4}^{•-}+S_{2}O_{8}^{2-}\;\to \;S_{2}O_{8}^{•-}+{\mathrm{SO}}_{4\;}^{2-}$$

(4) $${\mathrm{SO}}_{4}^{•-}+{\mathrm{SO}}_{4}^{•-}\;\to \;S_{2}O_{8}^{2-}$$

### 3.6. The Effect of Initial Solution pH

The influence of diverse initial solution pH levels on TC degradation is detailed in Figure 4f. In acidic conditions, the combined system showed better degradation performance, whereas in alkaline conditions, a lower TC degradation efficiency remained. The TC degradation efficiency obtained was 96.97% at a pH of 3, while the lowest degradation efficiency was recorded when the pH was 11 (50.6%). When the solution pH was below the equipotential point of Mn_1_-CN_1_ in the acidic environment, the Mn_1_-CN_1_ catalyst was positively charged [55]. This could promote the migration of photoexcited electrons to the catalyst surface and effectively lessen the compounding of e^−^ and h^+^, thus enhancing the catalytic efficiency of the catalyst. The Mn_1_-CN_1_ was negatively charged in an alkaline environment, which did not assist the transfer of photoexcited electrons to the catalyst surface, thus affecting photocatalytic efficiency.

Supplementary Figure S3 exhibits the UV-visible degradation spectra for each of the influencing factors. The intensity of the typical absorption peaks decreased as the photocatalytic reaction progressed. This indicated that the Mn_1_-CN_1_/PMS system produced effective active species. The reaction kinetic rate constant for TC degradation can be calculated from the first-order kinetic equation (Equation (5)), where *k* and *t* are the degradation rate constant and reaction time (min), respectively, *C_t_* is the TC concentration at t min (mg L^−1^), and *C*_0_ is the initial TC concentration (mg L^−1^).

(5) $$ln\left(C_{t}/C_{0}\right)=-kt$$

The photocatalytic process of MnO_2_/g-C_3_N_4_ was compared with other g-C_3_N_4_-based photocatalysts reported in the literature (Table 1). The findings proved that MnO_2_/g-C_3_N_4_ exhibited relatively better photocatalytic activity for organic pollutant removal. In addition, the kinetic diagrams at the TC level for each of the above influences are detailed as Supplementary Figure S4a–f, showing first-order reaction kinetics well explained the TC degradation rate, and the Mn_1_-CN_1_/PMS system had a high degradation rate constant (Supplementary Table S1).

**Figure 5:**
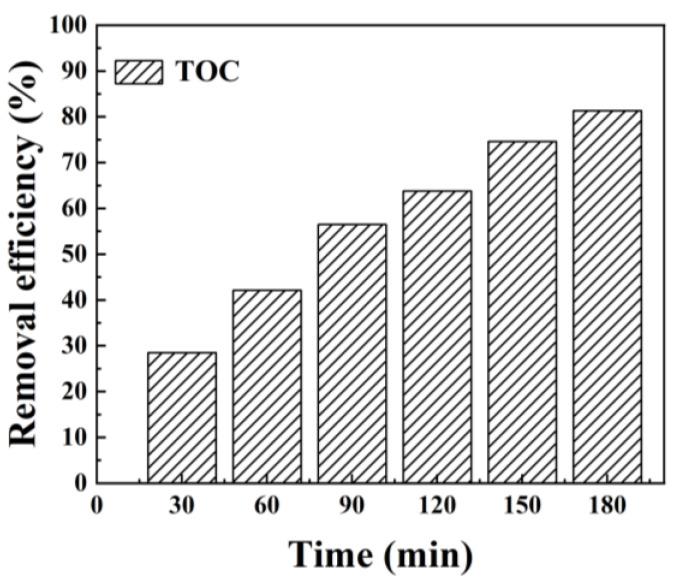
TOC removal by Mn_1_-CN_1_/PMS system for TC photocatalytic degradation. Reaction conditions: TC = 20 mg L^−1^; photocatalyst = 0.6 g L^−1^; PMS = 0.6 mM; T = 25 °C; and pH = 7.

The TOC analyzer (Analytickjena, multi N/C 2100s) was used to determine the change in total organic carbon in the TC solution. As shown in Figure 5, the removal of TOC increased with increasing of the photocatalytic treatment time. When the time was 180 min, the removal rate of TOC was 81.3%. This indicates that the Mn_1_-CN_1_/PMS system has significant photocatalytic treatment ability under visible light, which can oxidize industrial recalcitrant pollutants such as TC.

### 3.7. Stability of Photocatalytic Materials

**Figure 6:**
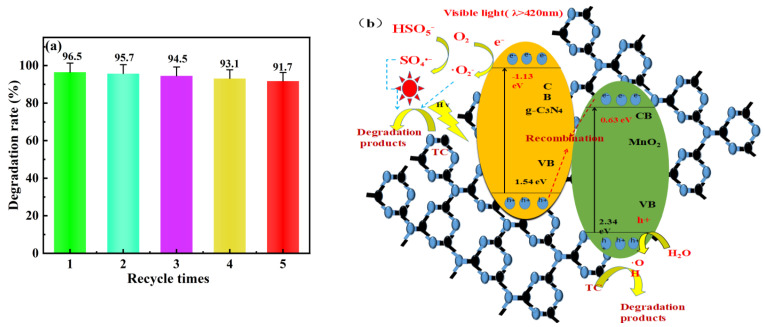
(**a**) Recyclability of Mn_1_-CN_1_ catalyst; (**b**) photocatalytic mechanism scheme of Mn_1_-CN_1_ composites.

As illustrated in Figure 6a, there was no appreciable difference between the first used catalyst and the five recycled ones, and TC degradation efficiency only declined from 96.5% to 91.7%. The decrease in TC degradation efficiency could be explained by the accumulation of intermediates on the Mn_1_-CN_1_ catalyst, which competed with the photocatalyst active site, and the loss of a small amount of photocatalyst during the cycling process experiments [63]. Meanwhile, to further confirm the stability, Mn_1_-CN_1_ was analyzed by SEM/TEM, XPS, and XRD after five cyclic experiments. The SEM/TEM image (Supplementary Figure S5) shows no great changes in Mn_1_-CN_1_ morphology. The XRD pattern (Supplementary Figure S6) shows that the diffraction peaks were significantly unchanged after the reaction. Furthermore, the XPS spectra of the recovered Mn_1_-CN_1_ were similar to those of the original catalyst, indicating that the surface structure of the sample did not change after five cycles of reaction (Supplementary Figure S7). The Mn_1_-CN_1_ composite photocatalyst was prepared with high stability, which is very useful for practical applications in wastewater treatment.

### 3.8. Visible Light Photocatalytic Degradation Mechanism

The active species trapping experiment was performed to investigate the active species involved in TC degradation [64,65]. Reagents IPA, BQ, EDTA-2Na, and MA were employed for capturing •OH, superoxide radical (•O_2_^−^), h^+^, and SO_4_^•−^, respectively. Supplementary Figure S8 shows the addition of IPA and MA significantly inhibited TC degradation efficiency, which diminished from 97% to 41.08% and 54.9%, correspondingly, revealing that •OH and SO_4_^•−^ were primary active species. Additionally, the presence of BQ also inhibited TC degradation efficiency, suggesting that •O_2_^−^ was also involved in the photocatalytic reaction. However, EDTA-2Na only slightly inhibited TC photodegradation, revealing that h^+^ was not the major active substance.

The possible mechanism of the degrading of TC pollutants by the Mn_1_-CN_1_/PMS system under visible light was investigated (Figure 6b). Unlike the type II configuration, the Z-type MnO_2_/g-C_3_N_4_ photocatalyst combines a semiconductor of high energy in the valence band (MnO_2_) with another semiconductor of lower bandgap and stronger reduction ability in the conduction band (g-C_3_N_4_). The electrons photogenerated in the MnO_2_ are cross-combined with the holes of the g-C_3_N_4_. This process yields the accumulation of separated charge carriers in the respective phases. The MnO_2_’s holes keep their strong oxidation ability, while the g-C_3_N_4_’s electrons retain their reduction potential. In Z-scheme heterojunction photocatalysts, the Mn_1_-CN_1_ generated photo-induced e^−^ and h^+^ (Equation (6)) were excited under visible light irradiation. Recombination reactions are conducted by photogenerated e^−^ in the CB of MnO_2_ and h^+^ in the VB of g-C_3_N_4_. The H_2_O or OH^−^ could be oxidized by h^+^ on the VB of MnO_2_ to produce •OH (Equations (7) and (8)). Photogenerated e^−^ on the CB of g-C_3_N_4_ reacted with dissolved oxygen, forming •O_2_^−^ (Equation (9)). Meanwhile, the PMS received photogenerated e^−^, which can be activated for producing SO_4_^•−^ (Equation (10)). The SO_4_^•−^ was able to react rapidly with OH^−^ for producing •OH (Equation (11)). The SO_4_^•−^ and •OH were vital for TC degradation (Equation (12)). Overall, ideal matching of the energy band structure between g-C_3_N_4_ and MnO_2_ can increase the amount of photogenerated e^−^ and h^+^ [66].

(6) $${\;\mathrm{MnO}}_{2}@g\text{-}C_{3}N_{4}+hv\;\to \;e^{-}+h^{+}$$

(7) $$H_{2}O+h^{+}\;\to \;\;•\mathrm{OH}+H^{+}$$

(8) $${\;\mathrm{OH}}^{-}+h^{+}\to •\mathrm{OH}$$

(9) $$O_{2}+e^{-}\to {•O}_{2}^{-}$$

(10) $$\mathrm{HS}O_{5}^{-}+e^{-}\to {\mathrm{OH}}^{-}+{\mathrm{SO}}_{4}^{•-}$$

(11) $${\mathrm{SO}}_{4}^{•-}+{\mathrm{OH}}^{-}\to •\mathrm{OH}+{\mathrm{SO}}_{4\;}^{2-}$$

(12) $$•\mathrm{OH}+{\mathrm{SO}}_{4}^{•-}+\mathrm{TC}\to \mathrm{Degradation}\;\mathrm{products}$$

**Figure 7:**
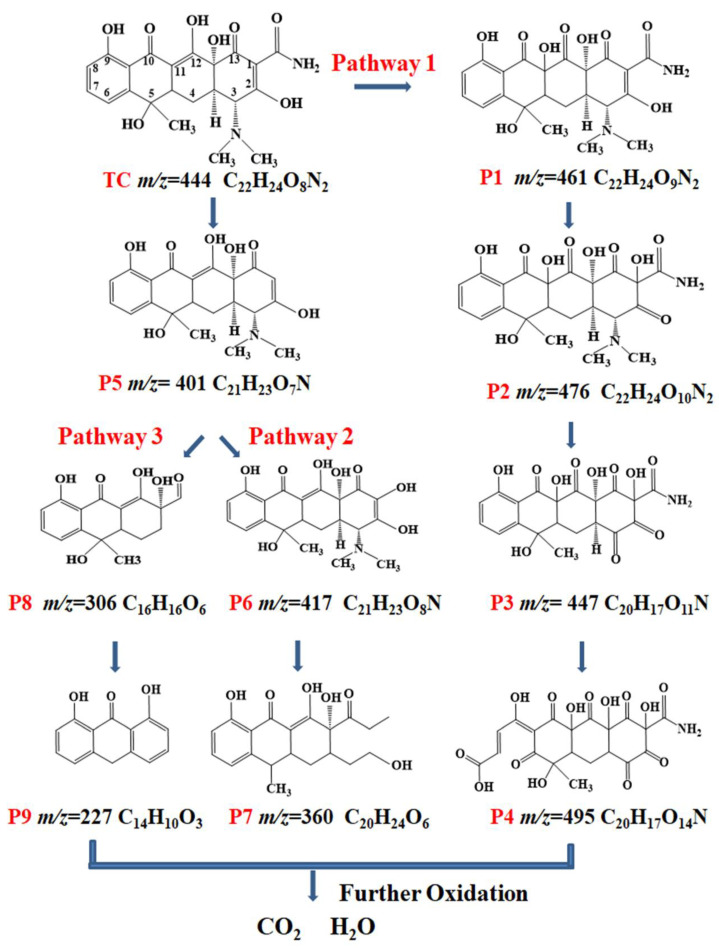
Three possible degradation pathways of TC in the Mn_1_-CN_1_/PMS system.

Intermediates of TC degradation were identified by HPLC/MS for future investigation of the TC degradation mechanism. More information on the nine main intermediates can be found in Supplementary Table S2. Three possible transformation reactions, hydroxylation, carboxylation, and N-C bond cleavage, are shown in Figure 7 [67,68,69]. Since -C=C- was conjugated to the oxygen on the adjacent -OH, P1 (*m*/*z* = 461) and P2 (*m*/*z* = 476) were derived from the hydroxyl addition of two enol groups. The -OH served as an electron-donating group, weakening the -C=C- linkage, and was attacked by •OH, generating ketone and epoxide group transformations. Meanwhile, P1 was susceptible to attack by reactive radicals and further formation of polyhydroxylated P2 (*m*/*z* = 476) because of low electron density of the C(1)=C(2) bond of P1. The C(3)-N bond of P2 was then oxidized for producing P3 (*m*/*z* = 447). Finally, P4 (*m*/*z* = 495) was obtained through ring-opening the aromatic ring on P3. In degradation pathway 2, the TC molecule underwent deamidation under the attack of reactive radicals for producing P5 (*m*/*z* = 401). P6 (*m*/*z* = 417) was formed by -OH addition to the deamidated C1. P7 (*m*/*z* = 360) was an intermediate product formed when -OH and -CH_3_ on C5 of P6 were attacked by h^+^ and •O_2_^−^ reactive oxide species, which were followed by dimethylamino and ring opening. In degradation pathway 3, the carbocyclic branched chain of P5 was destroyed by strong oxidizing radicals to produce P8 (*m*/*z* = 306). Finally, P8 underwent deacylation and demethylation dehydroxylation reactions to give P9 (*m*/*z* = 227). These intermediates were unstable and quickly oxidized to CO_2_ and H_2_O. A comparison of the by-products produced with the study of Yu et al. [28] revealed that the present study produced lesser quantities of by-products. This indicates that the Mn_1_-CN_1_/PMS system has significant photocatalytic treatment ability under visible light.

Supplementary Figure S9 shows Three-Dimensional Excitation-Emission Matrix (3DEEM) of TC at different photocatalytic times. The fluorescence peak of TC mainly occurred in the wavelength range of Ex/Em= (290–340 nm)/(380–460 nm), suggesting that TC was an aromatic protein-like compound. The intensity of the fluorescence peaks decreased with prolonged photocatalytic reaction time, suggesting that the aromatic benzene ring structure was damaged by the attack of the reactive species. The prominence peak almost disappeared after 180 min, suggesting that the simple aryl ring of TC was almost entirely disrupted (Supplementary Figure S8d).

### 3.9. TC Degradation Toxicity Analysis

**Figure 8:**
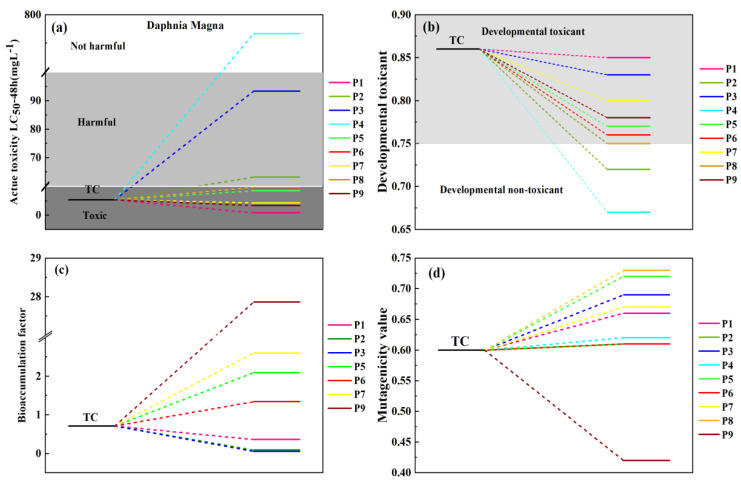
Toxicity analysis of reaction products: (**a**) The Daphnia magna LC_50_-48 h, (**b**) Developmental toxicity, (**c**) Bioaccumulation factors, (**d**) Mutagenicity.

To examine the environmental toxicity of TC, toxicity evaluation of the degradation products was used for contaminant status assessment. The biological toxicity of TC and its degradation intermediates were investigated via T.E.S.T. according to the quantitative structure–activity relationship (QSAR). Figure 8a,b shows the predicted values of daphnia magna LC_50_ and the developmental toxicity of the intermediates. The LC_50_-48 h values of most degradation intermediates were higher than that of TC, suggesting that TC was slowly transformed into less toxic intermediates. The products P2, P4, and P8 can be regarded as non-toxicants in Figure 8b. In addition, the results on the basis of bioaccumulation factors and mutagenicity illustrated that the photocatalytic reaction reduced the ecosystem hazard of TC and its intermediates [70]. Therefore, TC oxidation by Mn_1_-CN_1_/PMS system could reduce the toxicity of TC.

## 4. Conclusions

In this study, a Z-type Mn-CN heterojunction composite photocatalyst with excellent performance was successfully prepared by coupling the g-C_3_N_4_ with MnO_2_ via high-temperature thermal polymerization. The XRD, SEM, and TEM analysis showed synthesized Mn_1_-CN_1_ had good purity and a crystalline state. The catalytic experiments indicated that the Mn_1_-CN_1_/PMS system was the most effective, with the degradation efficiency of TC reaching 96.97% after 180 min of treatment, which was 38.65% higher relative to the g-C_3_N_4_/PMS system. The photocatalyst displayed marvelous stability and reusability after five cycle experiments. In addition, the PMS played an essential role in removing TC, while Mn_1_-CN_1_ was also an efficient activator for PMS under visible light irradiation. The active species trapping experiment showed •OH and ${\mathrm{SO}}_{4}^{•-}$ were major active species. Nine primary degradation intermediates and three possible degradation pathways were put forward. Toxicological evaluation of TC and its intermediates showed that the Mn_1_-CN_1_/PMS system could diminish TC toxicity. The findings showed the Mn_1_-CN_1_/PMS system was advantageous for the photocatalytic degradation of TC, which can offer potential application for the treatment of antibiotics in wastewater.

## Figures and Tables

**Table 1:** Comparison of TC degradation efficiencies with other C_3_N_4_-based photocatalysts.

Photocatalyst (g L^−1^)	TC Concentration (mg L^−1^)	Light Source Type	Volumes Tested	Time (min)	Removal (%)	References
AgCl/ZnO/g-C_3_N_4_ (1.0)	20 mg L^−1^	400–700 nm	40 ml	80 min	92.7%	[56]
Ag-C_3_N_4_/SnS_2_ (0.4)	15 mg L^−1^	500 W Xe lamp	50 ml	150 min	94.9%	[57]
g-C_3_N_4_/BiPO_4_ (1.0)	20 mg L^−1^	1000 W Xe lamp	50 ml	180 min	97%	[58]
C_3_N_4_/WO_3_ (0.4)	10 mg L^−1^	500 W Xe lamp (320–780nm)	100 ml	180 min	79.8%	[59]
Co_3_O_4_/g-C_3_N_4_ (0.4)	15 mg L^−1^	500 W Xe lamp (>420 nm)	100 ml	150 min	92.6%	[60]
g-C_3_N_4_/MnO_2_ /GO (0.5)	10 mg L^−1^	300 W Xe lamp (420 nm)	100 ml	90 min	91.4%	[61]
Ag_3_PO_4_/AgBr/g-C_3_N_4_(0.5)	40 mg L^−1^	300 W Xe lamp (>420 nm)	100 ml	25 min	80.2%	[28]
g-C_3_N_4_/Bi_2_WO_6_/AgI (0.6)	20 mg L^−1^	>420 nm	50 ml	60 min	91.1%	[62]
Mn_1_-CN_1_ (0.6)	20 mg L^−1^	300W xenon lamp	50 ml	180 min	96.97%	This study

## Data Availability

Data will be made available on request.

## Supplementary Materials

The following supporting information can be downloaded at: https://www.mdpi.com/article/10.3390/toxics12010070/s1, Figure S1: (a) Spectrogram of the xenon lamp light source BBZM−Ⅲ, (b) Diagram of the photo-catalytic device; Figure S2: the XPS valence band spectra; Figure S3: The UV absorption spectra of TC degradation under optimal reaction conditions for each influencing factor. (a) Mn1−CN1 photocatalyst, the amount of Mn1−CN1 photocatalyst dosed was 0.6 g L^–1^, the dosage of PMS was 0.6 mM; (b) The initial concentration of TC was 10 mg L^–1^; (c) The initial pH was 3; Figure S4: (a) Catalyst type on the reaction kinetics of TC; (b) Reaction kinetics of different ratios of Mn−CN catalysts for the degradation of TC; (c) Reaction kinetics of catalyst dosing for degradation of TC; (d) Reaction ki-netics of TC degradation at initial TC concentrations; (e) Kinetics of PMS dosage in response to TC; (f) Initial pH on the reaction kinetics of TC; Figure S5: SEM (a) and TEM (b) images of the Mn1−CN1 cycle after reaction; Figure S6: The XRD patterns of Mn1−CN1 before and after reaction; Figure S7: XPS spectra of Mn1−CN1 after reaction: full spectrum (a), C 1s (b), N 1s (c), Mn 2p (d), O1s (e); Figure S8: Active species trapping experiments of as−prepared Mn1−CN1 for TC degra-dation under visible light irradiation. [IPA, BQ, EDTA−2Na] = 1 mM L^–1^, [MA] = 10 mM L^–1^. The experimental conditions: [photocatalyst] = 0.6 g L^–1^, [TC] = 20 mg L^–1^, [PMS] = 0.6 mM, pH = 7, time=180 min, and T = 25 °C; Figure S9: 3DEEM spectra of TC solutions after different treatment times: (a) 0 min; (b) 60 min; (c) 120 min; (d) 180 min; Table S1: Reaction rate constants for each influencing factor.; Table S2: The TC degradation intermediates detected by HPLC−MS.
