# Supplementary material for: Z-Type Heterojunction MnO2@g-C3N4 Photocatalyst-Activated Peroxymonosulfate for the Removal of Tetracycline Hydrochloride in Water
Source: Toxics. 2024 Jan 14;12(1):70. doi: 10.3390/toxics12010070 (PMC10819820; doi:10.3390/toxics12010070)
Supplement: Supplementary file 1 [file toxics-12-00070-s001.zip › toxics-2740052-supplementary.pdf]

# Z-type heterojunction $MnO_2$@g-$C_3N_4$ photocatalyst-activated peroxymonosulfate for degradation of tetracycline hydrochloride in water

Guanglu Lu[1], Xinjuan Li[1], Peng Lu[1], He Guo[2], Zimo Wang[3], Qian Zhang[1], Yuchao Li[4], Wenbo Sun[1], Jiutao An[1*], Zijian Zhang[1*]

[1] College of Resources and Environment Engineering, Shandong University of Technology, Zibo 255000, China.

[2]Department of Environmental Engineering, College of Biology and the Environment, Nanjing Forestry University, Nanjing 210037, China.

[3] Department of Marine Engineering, Jimei University, Xiamen 361021, China.

[4] Research Institute of Clean Chemical Technology, School of Chemistry and Chemical Engineering, Shandong University of Technology, Zibo 255049, China

[*]Corresponding author: Prof.Jiutao An, E-mail address: anjiutao@163.com

Tel:+86-0533-2782219

Fax:+86-0533-2782219

**2 Texts**

**13 Pages**

**9 Figures**

**2 Table**

## Text S1. Materials

Melamine ($C_3H_6N_6$, purity 99%, 126.12) and tetracycline hydrochloride (TC,

$C_{22}H_{24}O_8N_2$·HCl, purity 96%, 480.9) were purchased from Aladdin Biochemical Technology, Shanghai, China. Potassium permanganate ($KMnO_4$, purity>99.5%, 158.03), manganese chloride ($MnCl_2$·$4H_2O$, purity>99%, 197.91), Potassium peroxymonosulfate ($KHSO_5$, purity 4.5% (active oxygen), 615.5), isopropyl alcohol (IPA, $C_3H_8O$, purity>99.9%, 60.01), benzoquinone (BQ, $C_6H_4O_2$, purity 99%, 108.09), Ethylenediamine- triacetate acid disodium salt (EDTA-2Na, $C_{10}H_{14}N_2O_8Na_2$·$2H_2O$, purity>99%, 372.24), and methanol (MA, $CH_4O$, purity 99.5%, 32.04) were supplied by Macklin Biochemical Technology (Shanghai, China). All of the above drugs and reagents are analytical grade and can be used directly without further purification.

**Text S2. Photocatalyst characterization**

The morphology and dimensions of the photocatalysts were identified using scanning electron microscopy techniques (SEM, FEI Sirion 200, Voltage: 200 kV), transmission electron microscopy (TEM, Tecnai G2 Spirit TWIN，Voltage: 220 kV), and high-resolution TEM (HRTEM). The elemental distribution was studied by X-ray energy spectrometry (EDS, Thermo Fisher, Quanta 250). The nitrogen adsorption-desorption isotherms recorded the pore size distribution and specific surface area at 77 K. X-ray diffraction (XRD) patterns were obtained using a Brucker D8 Advance diffraction apparatus (Germany) and Cu Kα radiation (λ=1.5418 Å, 2θ=10-70°). The surface chemistry of the samples was characterized by X-ray photoelectron spectroscopy (XPS Thermo Fisher ESCALAB XI+，Power:150 W) and Fourier transform infrared spectroscopy (FT-IR, Nicolet 5700, USA, λ=400-4000

cm$^{-1}$), and provided information on the chemical structure and functional groups. Solid-state UV-Vis diffuse reflectance spectroscopy (UV-Vis-DRS, PE Lambda 950, λ=200-800 nm) was used to study the optical properties and separation of photo-generated electrons and holes. Electrochemical impedance spectroscopy (EIS) was measured using a CHI 660B electrochemical system (Shanghai, China), the system is composed of an FTO covered with a Pt plate counter electrode, a photocatalyst working electrode, an Ag/AgCl reference electrode and an electrolyte of 0.5 M $Na_2SO_4$ solution. The degradation intermediates of TC were analyzed by HPLC-MS (Agilent/Bruker HP1100-HPLC/esquire2000, USA; Chromatographic conditions: injection volume 10.0 µL; column temperature 30 ℃; mobile phase: A: 0.1% formic acid aqueous solution; B: acetonitrile; flow rate: 0.35 mL/min; t: 20 min; mass spectrometry conditions: sheath gas rate: 40 arb; spray voltage: positive ions 3.8 kV, negative ions 2.8 kV; capillary temperature: 320 ℃, probe temperature: 300 ℃, the samples were filtered through a 0.22 µm microporous filter membrane and directly injected into the sample for LC-MS testing.). The molecular structural changes during TC degradation were analyzed using Three-Dimensional Excitation-Emission Matrix (3DEEM, Hitachi F7000, λ=200-900 nm, excitation sampling interval: 10 nm, scanning speed: 6000 nm/min, scanning interval: 1 nm).

The dominant wavelength of 400 nm and the 300-W solar simulator xenon lamp model is BBZM-Ⅲ. The manufacturer's stated spectrum is shown in figure S1. (a). Also, the photocatalytic device is shown in Figure S1. (b).

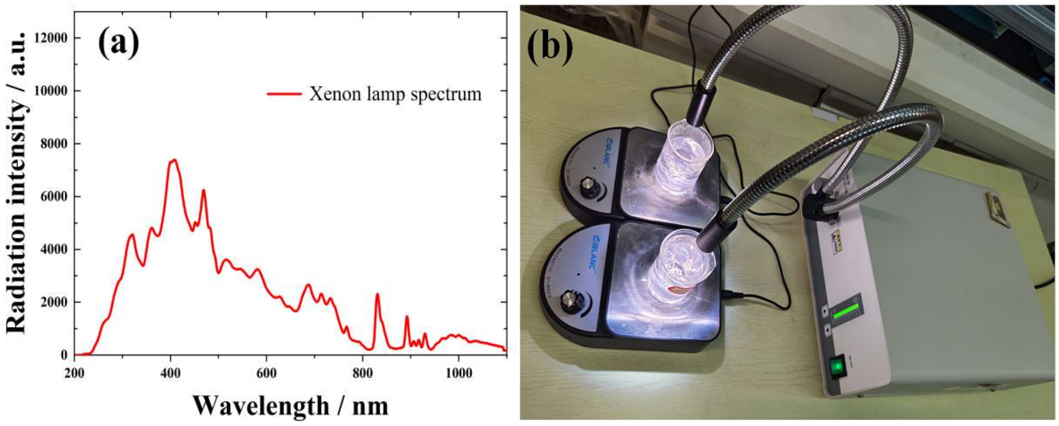


Fig.S1. (a) Spectrogram of the xenon lamp light source BBZM-Ⅲ; Fig. (b) Diagram of the photocatalytic device.

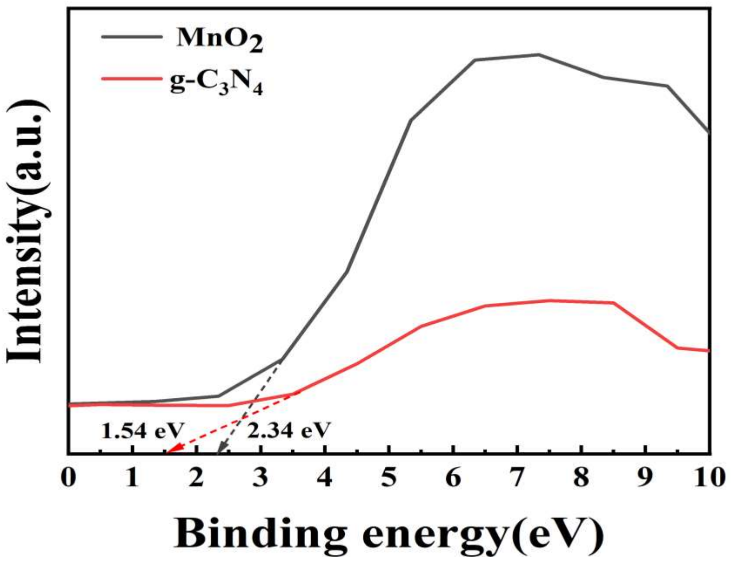


Figure.S2. the XPS valence band spectra

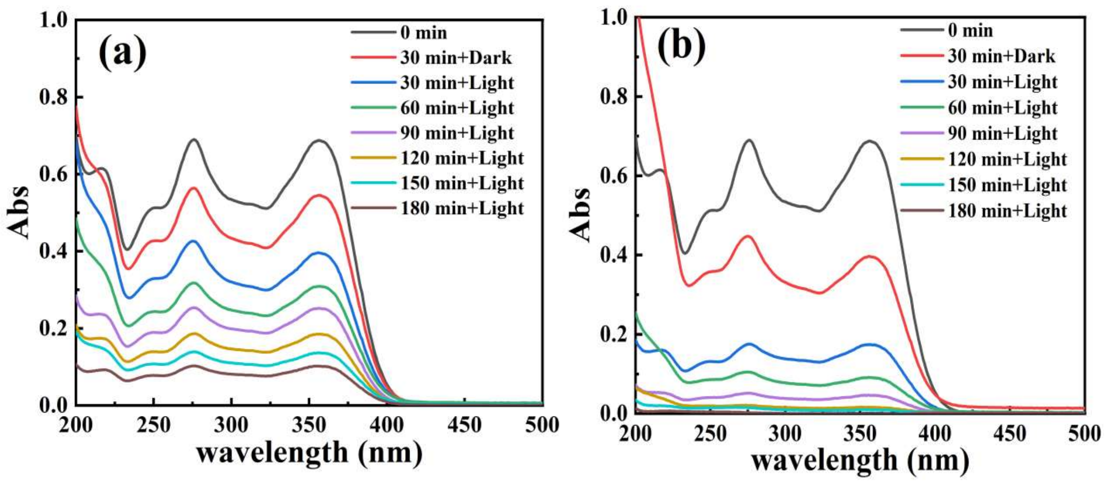

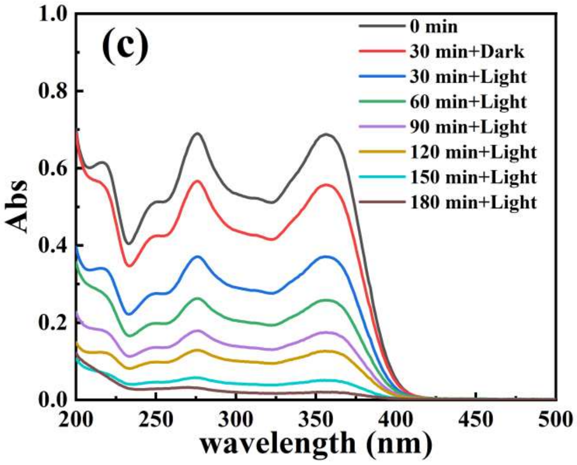


Figure.S3. The UV absorption spectra of TC degradation under optimal reaction conditions for each influencing factor. (a) $Mn_1$-$CN_1$ photocatalyst, the amount of $Mn_1$-$CN_1$ photocatalyst dosed was 0.6 g $L^{-1}$, the dosage of PMS was 0.6 mM; (b) The initial concentration of TC was 10 mg $L^{-1}$; (c) The initial pH was 3.

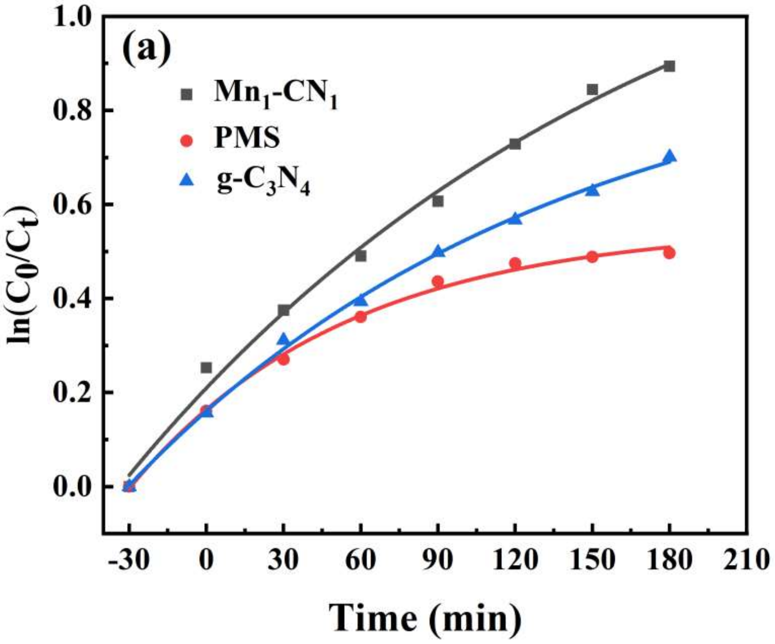

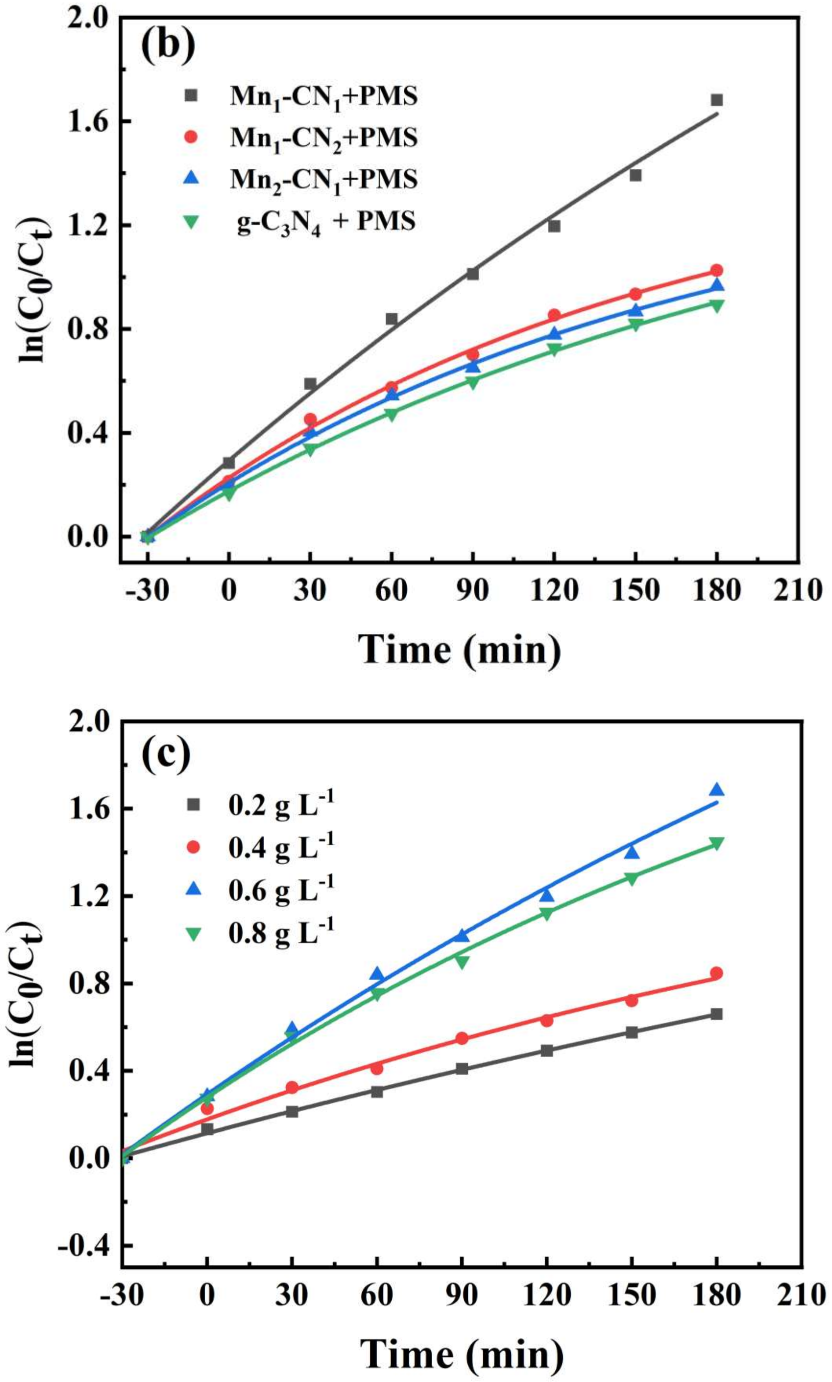
(b)
Mn1-CN1+PMS
Mn1-CN2+PMS
Mn2-CN1+PMS
g-C3N4 + PMS
ln(C0/Ct)
Time (min)
(c)
0.2 g L-1
0.4 g L-1
0.6 g L-1
0.8 g L-1
ln(C0/Ct)
Time (min)

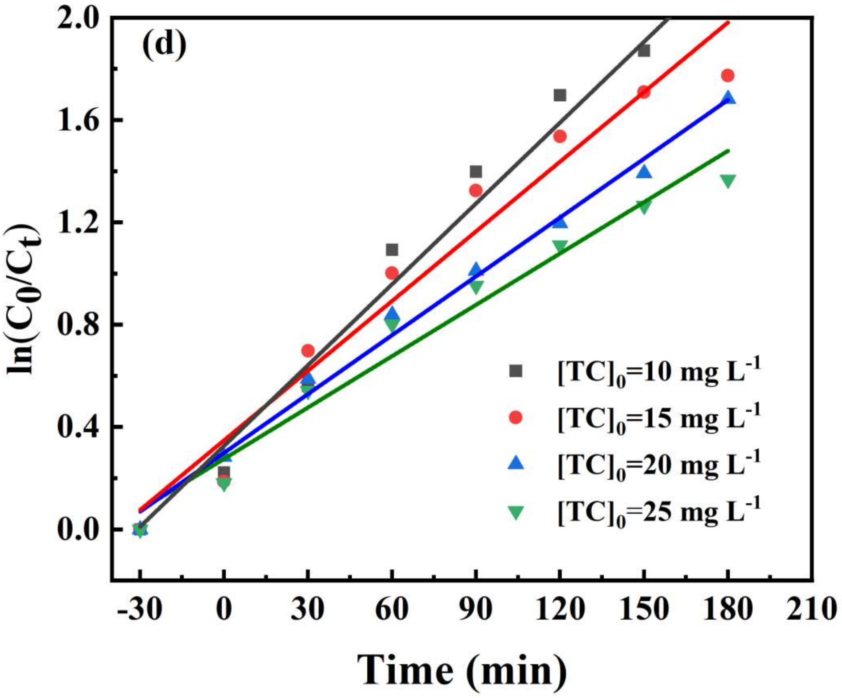
(d)
ln(C0/Ct)
Time (min)
[TC]0=10 mg L-1
[TC]0=15 mg L-1
[TC]0=20 mg L-1
[TC]0=25 mg L-1

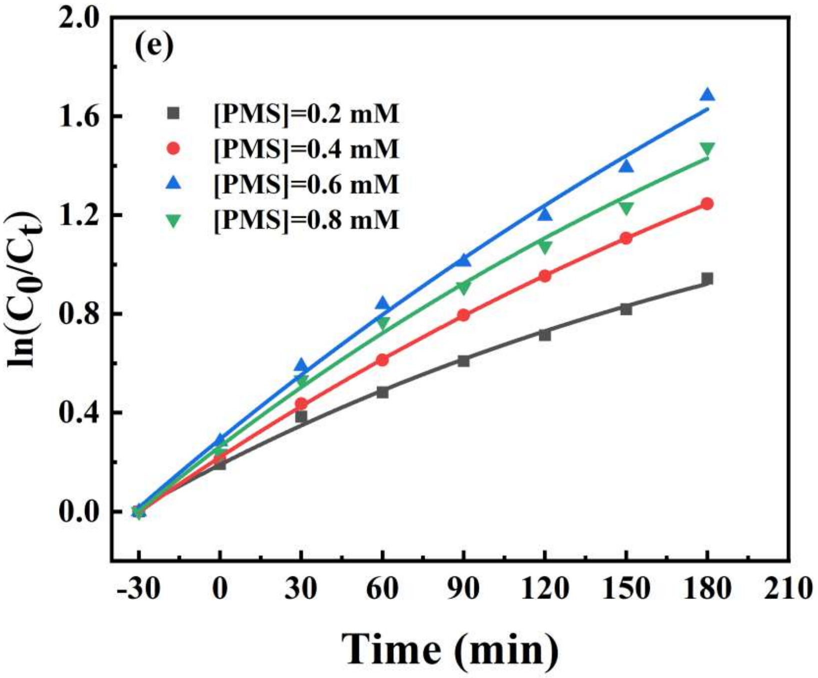
(e)
ln(C0/Ct)
Time (min)
[PMS]=0.2 mM
[PMS]=0.4 mM
[PMS]=0.6 mM
[PMS]=0.8 mM

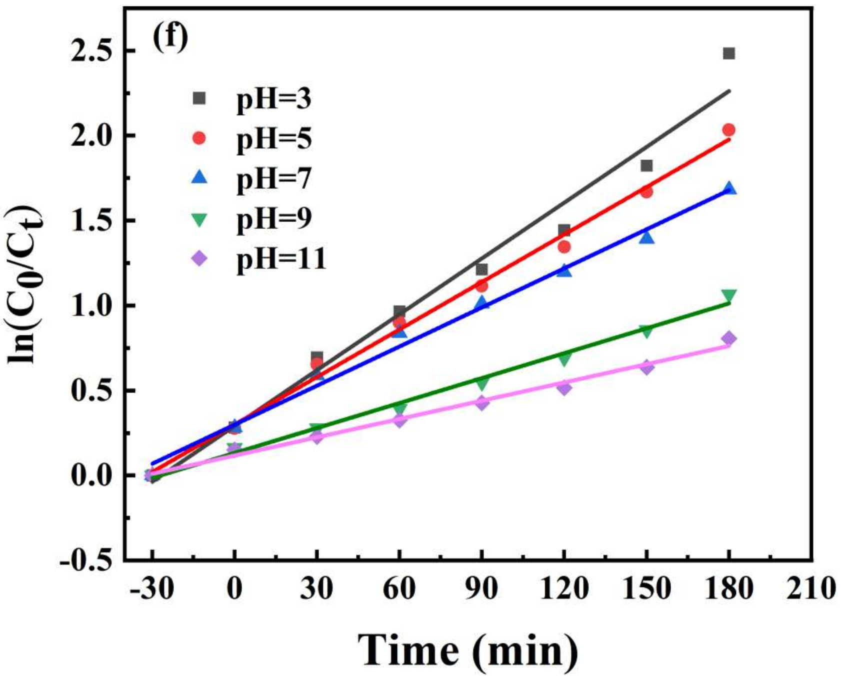


Figure.S4. (a) Catalyst type on the reaction kinetics of TC; (b) Reaction kinetics of different ratios of Mn-CN catalysts for the degradation of TC; (c) Reaction kinetics of catalyst dosing for degradation of TC; (d) Reaction kinetics of TC degradation at initial TC concentrations; (e) Kinetics of PMS dosage in response to TC; (f) Initial pH on the reaction kinetics of TC.

Table S1. Reaction rate constants for each influencing factor.

| | | | | |
|---|---|---|---|---|
| Catalysts | $Mn_1$-$CN_1$ | PMS | g-$C_3N_4$ | |
| k | 0.0486 | 0.01193 | 0.006 | |
| $R^2$ | 0.99421 | 0.99687 | 0.99828 | |
| Loading of $MnO_2$ | $Mn_1$-$CN_1$+PMS | $Mn_1$-$CN_2$+PMS | $Mn_2$-$CN_1$+PMS | g-$C_3N_4$+PMS |
| k | 0.0215 | 0.00553 | 0.00515 | 0.004 |
| $R^2$ | 0.99525 | 0.99787 | 0.99878 | 0.99946 |
| The dosage of | 0.2 g $L^{-1}$ | 0.4 g $L^{-1}$ | 0.6 g $L^{-1}$ | 0.8 g $L^{-1}$ |

| | | | | | |
|---|---|---|---|---|---|
| $Mn_1$-$CN_1$ | | | | | |
| k | 0.00152 | 0.00299 | 0.0215 | 0.00335 | |
| $R^2$ | 0.99855 | 0.99013 | 0.99525 | 0.99817 | |
| Initial concentration of TC | 10 mg $L^{-1}$ | 15 mg $L^{-1}$ | 20 mg $L^{-1}$ | 25 mg $L^{-1}$ | |
| k | 0.01053 | 0.00908 | 0.00766 | 0.00669 | |
| $R^2$ | 0.98067 | 0.96062 | 0.99142 | 0.96939 | |
| PMS dosage | 0.2 mM | 0.4 mM | 0.6 mM | 0.8 mM | |
| k | 0.00149 | 0.00273 | 0.00416 | 0.00284 | |
| $R^2$ | 0.99659 | 0.99987 | 0.99525 | 0.99497 | |
| Initial pH | 3 | 5 | 7 | 9 | 11 |
| k | 0.01095 | 0.00932 | 0.00766 | 0.00489 | 0.00358 |
| $R^2$ | 0.97862 | 0.99454 | 0.99142 | 0.99266 | 0.99067 |

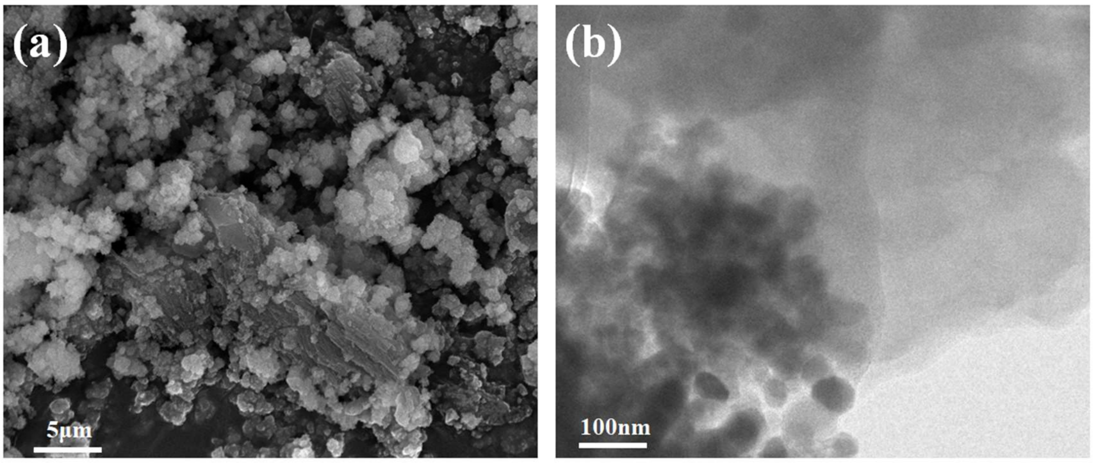


Fig.S5. SEM (a) and TEM (b) images of the $Mn_1$-$CN_1$ cycle after reaction.

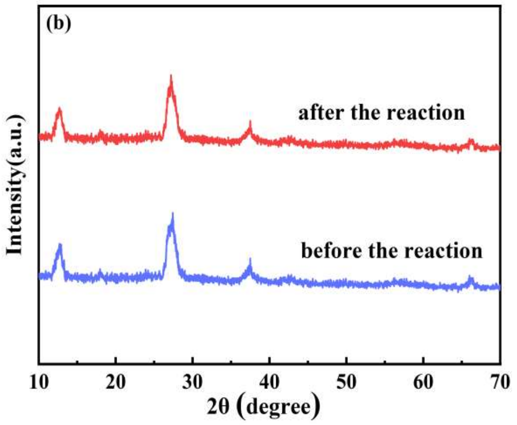


Figure.S6. The XRD patterns of $Mn_1$-$CN_1$ before and after reaction;

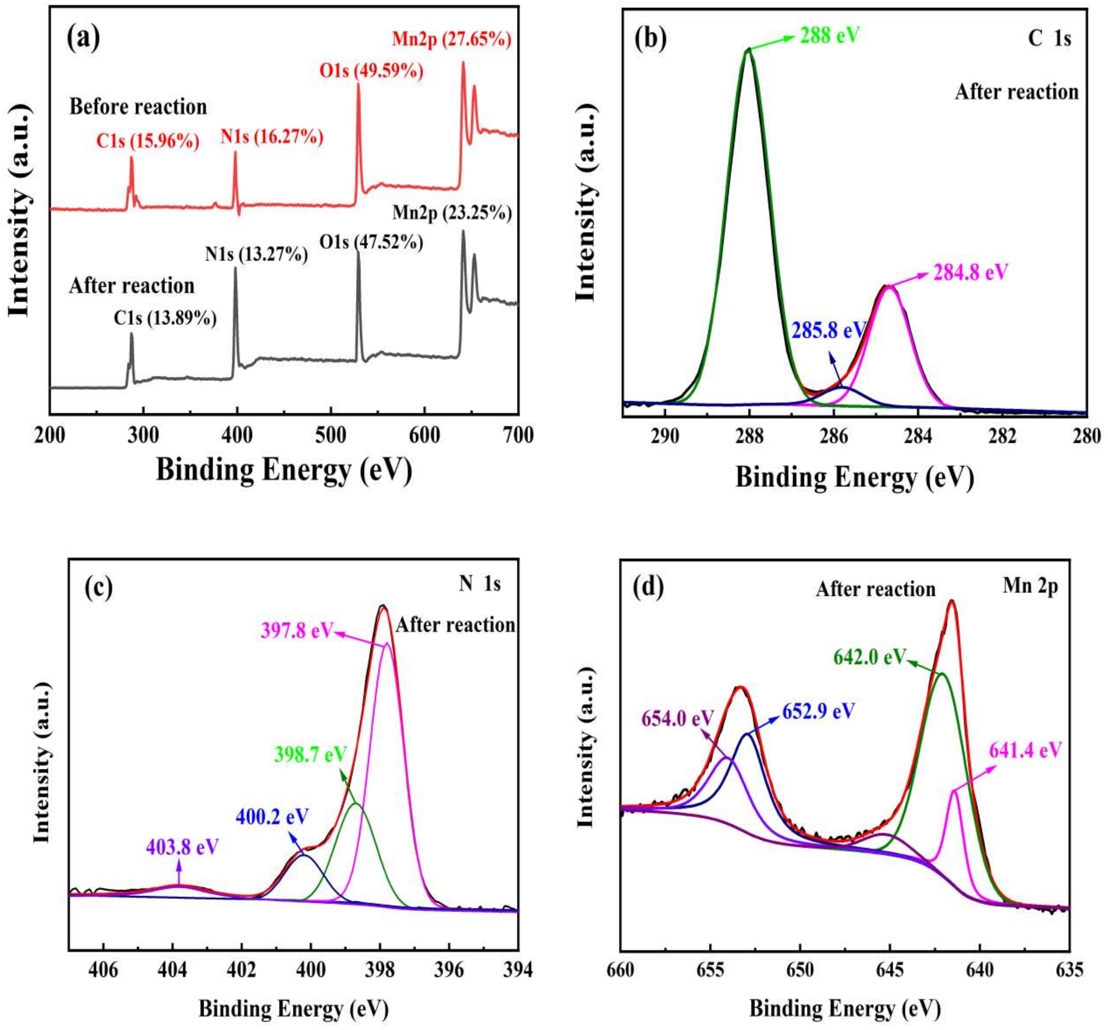

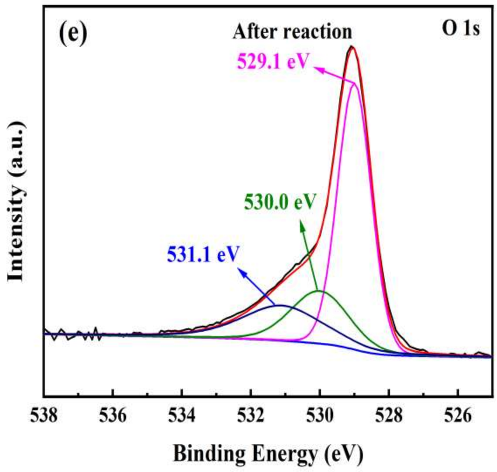


Figure.S7. XPS spectra of $Mn_1$-$CN_1$ after reaction: full spectrum (a), C 1s (b), N 1s (c), Mn 2p (d), O1s (e).

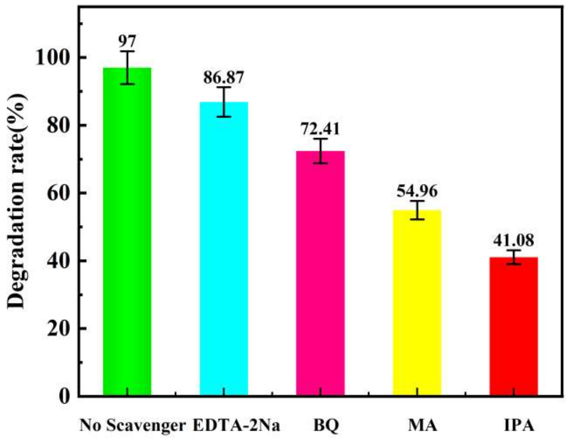


Figure.S8. Active species trapping experiments of as-prepared $Mn_1$-$CN_1$ for TC degradation under visible light irradiation. [IPA, BQ, EDTA-2Na]=1 mM $L^{-1}$, [MA] = 10 mM $L^{-1}$. The experimental conditions: [photocatalyst] = 0.6 g $L^{-1}$, [TC] = 20 mg $L^{-1}$, [PMS] = 0.6 mM, pH = 7, time=180 min, and T = 25 ℃.

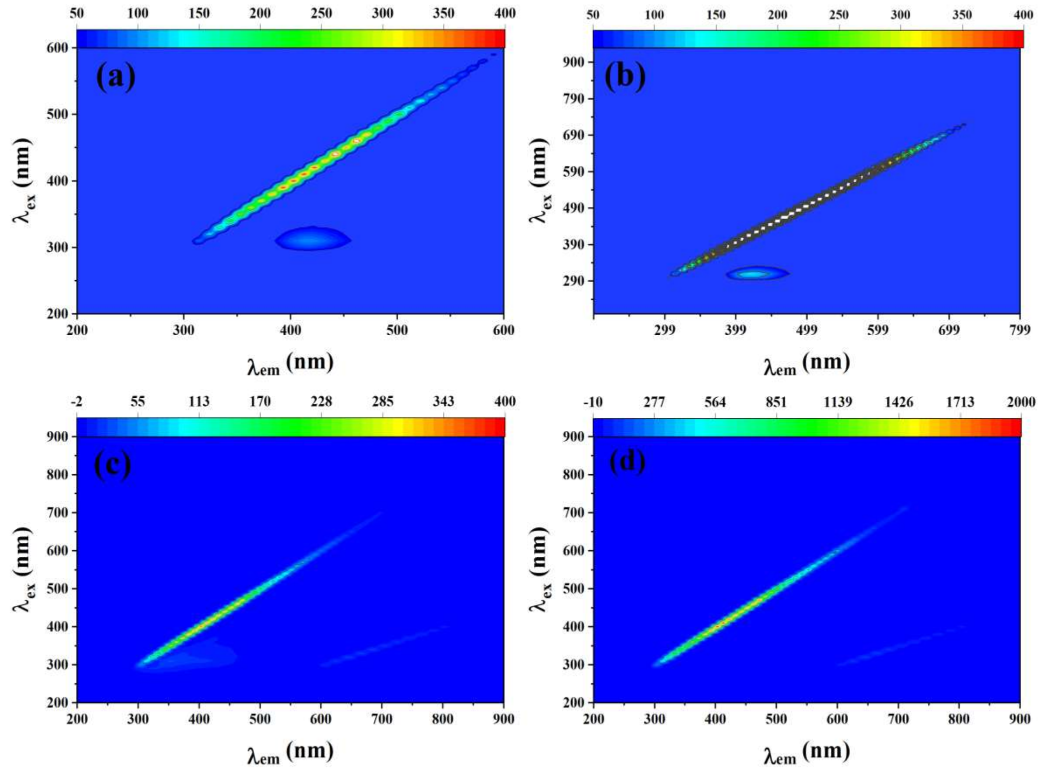


Figure.S9. 3DEEM spectra of TC solutions after different treatment times: (a) 0 min; (b) 60 min; (c) 120 min; (d) 180 min.

Table S2. The TC degradation intermediates detected by HPLC-MS.

| No. | m/z | Molecular formula | Chemical structure |
|---|---|---|---|
| 1 | 461 | $C_{22}H_{24}N_2O_9$ | 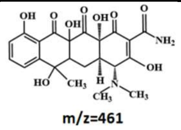  |
| 2 | 476 | $C_{22}H_{24}N_2O_{10}$ | 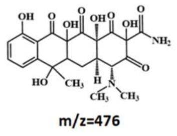  |
| 3 | 447 | $C_{20}H_{17}NO_{11}$ | 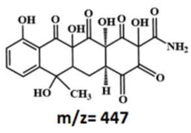  |
| 4 | 495 | $C_{20}H_{17}NO_{14}$ | 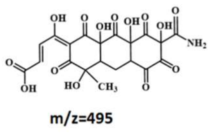  |

| | | | |
|---|---|---|---|
| TC | 444 | $C_{22}H_{24}N_2O_8$ | 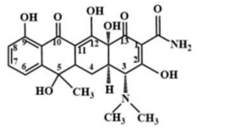 m/z=444 |
| 5 | 401 | $C_{21}H_{23}O_7N$ | 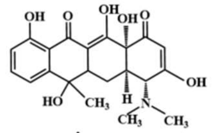 m/z= 401 |
| 6 | 417 | $C_{21}H_{23}O_8N$ | 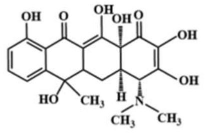 m/z=417 |
| 7 | 360 | $C_{20}H_{24}O_6$ | 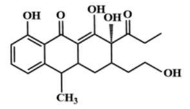 m/z=360 |
| 8 | 306 | $C_{16}H_{16}O_6$ | 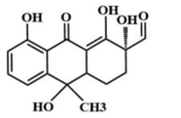 m/z=306 |
| 9 | 227 | $C_{14}H_{10}O_3$ | 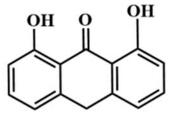 m/z=227 |
